# Immunolabelling and Micro‐Computed Tomography Revealed Age‐Related Alterations in 3D Microvasculature of Tendons

**DOI:** 10.1111/acel.70293

**Published:** 2025-11-18

**Authors:** Nodoka Iwasaki, Jack Llewellyn, Jeanne Brown, Danae E. Zamboulis, Elizabeth J. T. Finding, Caroline P. D. Wheeler‐Jones, Chavaunne T. Thorpe

**Affiliations:** ^1^ Comparative Biomedical Sciences Royal Veterinary College London UK; ^2^ Department of Clinical Sciences, School of Veterinary Medicine Aristotle University of Thessaloniki Thessaloniki Greece

**Keywords:** 3D imaging, ageing, immunolabelling, interfascicular matrix, micro‐computed tomography, microvasculature, tendon

## Abstract

Tendon degeneration is common, and its risk increases with age both in humans and horses. Tendon regeneration and healing is limited due to inherent low cell density and vascularisation, and current treatments are insufficient as indicated by scar tissue formation and a high re‐injury rate. The tendon vasculature plays a crucial role in tendon homeostasis, regeneration and healing, making it a potential therapeutic target. However, the effect of ageing on the tendon microvasculature is poorly understood. Here, we provide the first comprehensive characterisation of the tendon microvasculature. We employed high‐resolution 3D imaging techniques, using micro‐computed tomography (μCT) and confocal microscopy, to investigate age‐related alterations in the vasculature within the equine superficial digital flexor tendon (SDFT), a functional equivalent of the human Achilles tendon. μCT analysis revealed a well‐developed vascular network within the interfascicular matrix (IFM) and demonstrated significant age‐associated reductions in vascular volume (70%), vessel diameter (30%) and density (74%). 3D immunolabelling showed significant reductions in MYH11‐ (96%) and desmin‐positive (78%) volumes; however, there was a pronounced age‐associated increase in von Willebrand factor (VWF)‐positive volume (220%), which was accompanied by a significantly higher (249%) pericyte density. Taken together, these results indicate a loss of larger blood vessels in the IFM but an increase in small vessel formation, suggesting that neo‐angiogenesis is induced in aged tendon alongside a loss of vascular homeostasis. These insights enhance our understanding of tendon ageing and may contribute to developing new therapeutic approaches for improving tendon health and repair in older individuals.

## Graphical abstract

Immunolabelling and μCT imaging reveal that ageing significantly reduces tendon vascular volume, density and smooth muscle coverage while increasing endothelial cell and pericyte density, indicating a loss of larger vessels alongside enhanced neo‐angiogenesis and disrupted vascular homeostasis, which may contribute to impaired tendon regeneration and repair in ageing.

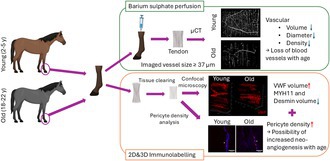

## 1 Introduction

Tendon injuries are common; 32 million musculoskeletal injuries are reported in the United States every year, of which 30%–45% involve tendons or ligaments (Butler et al. 2004; Ponkilainen et al. 2022; Yang et al. 2013) and ageing is one of the main risk factors for tendon degeneration (McCarthy and Hannafin 2014; Riasat et al. 2020; Teunis et al. 2014). It is well established that ageing has wide‐ranging effects on tendon, altering tendon structure, composition, turnover and mechanical properties (Boros and Freemont 2017; Godinho et al. 2017, 2021; O'Brien et al. 2021; Pardes et al. 2017; Peffers et al. 2014; Thorpe et al. 2010, 2016, 2017). While the vasculature is important in tendon homeostasis, regeneration and healing (Docheva et al. 2015; Fenwick et al. 2002), the structure of tendon vasculature has not been described in detail. Further, few studies have investigated how the tendon vasculature is affected by ageing, and those that have report contradictory findings, with some showing no changes in vascular density with age (Gillis et al. 1997), and others demonstrating a significant reduction in vessel density (Brewer 1979; Márquez‐Arabia et al. 2017). In these latter studies, vascular density was assessed in two‐dimensional (2D) histological sections, which may have contributed to these contradictory findings. This highlights the importance of employing state‐of‐the‐art approaches, such as 3D imaging, to comprehensively investigate age‐related vascular changes.

Tendons are dense, highly organised connective tissues that link muscles to bones, facilitating the transfer of muscle‐generated forces to the skeletal system (Im and Kim 2020), and therefore play a crucial role in joint movement and maintaining body posture (Bianchi et al. 2021). Structurally, tendons exhibit a hierarchical organisation consisting of collagen fibrils, fibres, fascicles and fascicle bundles. Fascicles and fascicle bundles are enclosed by the interfascicular matrix (endotenon), a connective tissue layer that contains blood vessels, lymphatics and nerves (Elliott 1965; Kannus 2000). Tendon is less vascularised than muscle and bone and receives blood from three main areas: the muscle‐tendon junction (MTJ), the osteotendinous junction (enthesis) and the paratenon (Ahmed et al. 1998). While tendon has historically been referred to as relatively avascular (Ahmed et al. 1998; Schmidt‐Rohlfing et al. 1992), the interfascicular matrix (IFM) is rich in blood vessels, as evidenced by enrichment of vascular proteins, CD146 (also known as MCAM) and large numbers of vascular cells in this region (Marr et al. 2023; Zamboulis et al. 2024).

Tendon healing is slow and inefficient, a limitation that is further exacerbated in aged individuals, and which is thought to be due to low cell density and limited vascularisation (Docheva et al. 2015; Heinemeier et al. 2013; Korcari et al. 2023; Liu et al. 2011). With ageing, tendons exhibit declines not only in their mechanics, structure, composition and cellular activity but also in their response to injury and overall healing capacity, and they become more prone to injuries and develop tissue defects (Ackerman et al. 2017; Korcari et al. 2023; Teunis et al. 2014; Yasui et al. 2017). Ageing also leads to impaired angiogenesis and heightens the risk of processes associated with pathological vessel formation in various tissues (Lähteenvuo and Rosenzweig 2012). However, the mechanisms underlying age‐related impairments in angiogenesis remain poorly understood, further complicated by cellular dynamics that vary across distinct regions of the microvascular network and between different tissue types (Hodges et al. 2018). Despite the critical role of vascularisation in tissue repair, the impact of ageing on tendon microvasculature and its consequences for tendon healing have not yet been investigated. Understanding these changes could reveal new therapeutic opportunities to enhance tendon regeneration and repair, thereby overcoming the limitations of current interventions such as scar tissue formation and high rates of reinjury (Bianchi et al. 2021). Consequently, a deeper understanding of the influence of ageing on tendon vasculature is required to develop novel therapeutic strategies to treat age‐related tendon degeneration.

Obtaining young, healthy human tendon is challenging, so appropriate models are needed to study the effects of ageing in healthy tendon. The horse represents an excellent model for studying tendon because the forelimb superficial digital flexor tendon (SDFT) is structurally and functionally similar to the human Achilles tendon. Both are energy‐storing tendons with a similar hierarchical structure, share comparable epidemiology and aetiology of disease, and exhibit similarly poor healing following injury (Innes and Clegg 2010; Patterson‐Kane and Rich 2014). Horse tendons are subjected to high mechanical loads during exercise, comparable to those experienced by tendons in human athletes, and often naturally suffer from injury during training and racing (Crevier‐Denoix et al. 1997; Dowling et al. 2000; Patterson‐Kane and Rich 2014; Shojaee 2023). This makes the horse tendon a valuable model for studying human tendon pathology.

This study aimed to comprehensively characterise age‐related vasculature alterations in the equine SDFT using high‐resolution 3D imaging techniques: μCT and confocal microscopy. These advanced imaging methods have enabled us to quantify the vascular alterations associated with ageing in tendons. This knowledge may be useful in efforts to develop new therapeutics for boosting tendon regeneration and repair across species.

## 2 Materials and Methods

### 2.1 Single Cell RNA Sequencing Data Analysis

Single cell RNA sequencing (scRNA‐seq) data from young (*n* = 4; age: 3–4 years) and old (*n* = 4; age: > 17 years) SDFTs obtained from a previous project, without any evidence of pathology (Zamboulis et al. 2024) were re‐analysed using Seurat (v4.1.1) in R Studio (v2021.09.2) to identify vascular gene expression in SDFTs, which was not reported in detail in the previous study. The FindCluster function was used to identify cell clusters (0.6 resolution) and differentially expressed (DE) genes (*p* < 0.05, adjusted *p*‐value using Bonferroni correction applied) between young and old SDFTs, which were analysed using the FindAllMarkers function to identify markers specific for endothelial cell, mural cell (vascular smooth muscle cells and pericytes) and tenocyte clusters.

### 2.2 Sample Acquisition

Forelimbs, distal to the carpus, were collected from eight young (age: 2–5 years) and eight aged (age: 18–22 years) Thoroughbred or Thoroughbred‐type horses with absence of clinical disease euthanised at a commercial abattoir for reasons unrelated to this project. The SDFT has been shown to reach maturity by the age of 2 years (Birch et al. 1999), and horses are considered geriatric above the age of 15 (Ireland et al. 2011). The samples were obtained from both male and female horses. Sample collection was approved by the Royal Veterinary College's Clinical Research Ethical Review Board (URN 2021 2077‐2). All samples used in this study were obtained from the mid‐metacarpal region of the SDFT, the region most susceptible to injury. Following dissection, specimens were examined macroscopically to ensure that they were free from signs of injury.

### 2.3 2D Immunofluorescent Labelling/Immunohistochemistry

Forelimbs were stored at 4°C overnight and the SDFTs from young horses (*n* = 4) were harvested the following day. Pieces of SDFT were snap frozen in hexane cooled on dry ice, and then embedded using OCT (15212776, Fisher Scientific, MA, USA). SDFT cryosections (10 μm thickness) were fixed in 4% paraformaldehyde (PFA) for staining using cell surface marker antibodies (CD144 and PDGFRB), and an ice‐cold methanol/acetone solution (1:1) was used for other antibodies for 10 min at room temperature. Non‐specific binding of antibodies was blocked by incubating samples with 5% goat serum (GS; ab7481, Abcam, Cambridge, UK) in Tris‐buffered saline (TBS) for 1 h at room temperature. The samples were incubated with primary antibodies in 5% GS (details shown in Table 1) for 2 h at room temperature. Some of the primary antibodies were chosen based on their use in previous equine studies (Finding et al. 2024; Zamboulis et al. 2024). After washing twice with TBS, the sections were incubated with secondary antibodies (Fisher Scientific, MA, USA) in 5% GS (1:400, details shown in Table 1) for 1 h at room temperature. The sections were then incubated with DAPI (0.1 μg/mL) for 10 min at room temperature, followed by two washes in TBS. The sections were mounted with ProLong Gold Antifade Mountant (P10144, Fisher Scientific, MA, USA) and allowed to cure for 2–3 h before imaging using an Eclipse Ni‐E upright microscope (Nikon Instruments Inc., Tokyo, Japan).

**TABLE 1:** Details of primary and secondary antibodies used in 2D immunofluorescent labelling.

Primary antibody	Company	Cat. no.	Dilution	Fixative	Secondary antibody	Target
CD144 / VE‐Cadherin	Thermo Scientific	MA5‐28541	1 in 100	4% PFA	Anti‐mouse AF594	Endothelial cells
CD31 / PECAM1	Abcam	ab28364	1 in 50	4% PFA	Anti‐rabbit AF594	Endothelial cells
Von Willebrand factor (VWF)	Dako	A0082	1 in 150	Methanol/acetone	Anti‐rabbit AF594	Endothelial cells
ETS‐related gene (ERG)	Abcam	ab214341	1 in 50	Methanol/acetone	Anti‐mouse AF594	Endothelial cells
Desmin	Dako	M0760	1 in 50	Methanol/acetone	Anti‐mouse AF488	Smooth muscle cells
Myosin‐11 (MYH11)	Thermo Scientific	PA5‐82526	1 in 200	Methanol/acetone	anti‐rabbit AF488	Smooth muscle cells
PDGFRB	Abcam	ab69506	1 in 50	4% PFA	Anti‐rabbit AF488	Pericytes

### 2.4 Barium Sulphate Perfusion of Equine Limbs

The protocol for perfusion was adapted from a previous study (Liu et al. 2019). Immediately after euthanasia and disarticulation of the limb at the level of the carpus, the distal limbs from young and old horses (*n* = 4 per age group) were perfused first with physiological saline (0.9% NaCl in DI water) and then with heparinised saline (50 IU/mL in physiological saline) (H5515, Sigma‐Aldrich, MA, USA) through the median artery in the digital tendon sheath using a 16‐gauge needle (Amazon, WA, USA) attached to a 50 mL syringe (613‐6079, VWR, PA, USA). Perfusion was repeated until the fluid exiting the veins became clear. The limbs were transported to the Royal Veterinary College and stored at 4°C overnight. The following day, the limbs were perfused with physiological saline to remove any residual blood, both before and after a 45‐min incubation at 50°C in a water bath to heat the SDFT to approximately 37°C. The limbs were then perfused with barium sulphate gelatine solution (20 g of barium sulphate, 222510010, Fisher Scientific, MA, USA) and 5 g of gelatine (G1890, Sigma‐Aldrich, MA, USA) in 100 mL of physiological saline, heated to 37°C, using a 16‐gauge needle and 50 mL syringe. The limbs were stored in a cold room overnight to allow the gelatine to solidify, and the SDFTs were harvested the following day. SDFTs were each cut into 15 mm length pieces and then fixed in 4% PFA for 24 h. One 15 mm piece from the mid‐metacarpal region of each SDFT was imaged using μCT.

### 2.5 μCT Imaging

Barium sulphate‐perfused SDFTs were wrapped in cling film to avoid dehydration and placed in a tube to prevent the sample from moving during imaging. A Skyscan 1172F (version 1.5, Skyscan, Kontich, Belgium) was used with an X‐ray source at 50 kV tube voltage and 200 μA tube current with 960 ms exposure time. The voxel size was 10 μm, and 180° scans were performed with a 0.5 mm Aluminium filter, frame averaging at 2, and with a rotation step of 0.4°. Slice reconstruction was performed using NRecon (version 1.7.1.0). The reconstructed images were segmented to remove the epitenon (Figure S1) and analysed using CTAn (version 1.17.7.1). Vascular volume and total tissue volume were quantified using CTAn, allowing calculation of the percentage of vascular volume relative to the entire tissue volume. Whole tissue volume was defined by applying a threshold distinct from that used to isolate the vasculature. The mean vascular diameter (average diameter of all vessels within the region of interest) and the degree of anisotropy, indicating the level of vessel orientation, were also measured using CTAn. The vascular density, defined as the number of vessels per mm^2^, was measured by counting the number of vessels in 2D cross‐sectional images and averaging the vascular densities in 3 images from the top, middle and bottom portions of each reconstructed dataset. CTVox (version 3.3.0) was used to visualise the 3D reconstructed images, and Avizo (Avizo 2021.1, ThermoFisher Scientific, Waltham, MA, USA) was used to apply colour mapping to the images based on vascular diameter.

### 2.6 3D Immunolabelling

Forelimbs from young and old horses (*n* = 4; each age group) were stored at 4°C overnight and the SDFTs were harvested the following day and fixed in 4% PFA for 24 h as detailed above. The fixed tissues were cut into 5 mm cubes for 3D immunolabelling, using a protocol adapted from a previous study (Marr et al. 2020). Permeabilisation was performed using 50% (v/v) methanol:TBS, 80% (v/v) methanol:dH_2_O, and 100% methanol for 2 h, and 20% (v/v) dimethylsulphoxide (DMSO):methanol, 80% (v/v) methanol:dH_2_O, 50% (v/v) methanol:TBS for 30 min at 4°C, respectively, with gentle shaking. The samples were stored in TBS overnight at 4°C. Blocking was performed using blocking solution (0.2% Triton X‐100, 6% donkey serum, 6% GS, 10% DMSO in TBS) for 72 h at 37°C with gentle shaking. The samples were then incubated with primary antibody (diluted in blocking solution, details shown in Table 2) for 72 h at 37°C with gentle shaking, followed by 5 washes with 0.2% Tween‐20 in TBS for 1 h each at room temperature. The samples were incubated with secondary antibody (diluted in blocking solution, details shown in Table 2) for 24 h at 37°C with gentle shaking, followed by 5 washes with 0.2% Tween‐20 in TBS for 1 h at room temperature. Samples were then incubated in DAPI solution (5 μg/mL in TBS) at 4°C overnight. Samples were dehydrated as described above with increasing concentrations of methanol. Two‐step tissue clarification was performed by immersing samples in Visikol HISTO‐1 (H1‐30, Sigma‐Aldrich, MA, USA) for 24 h, followed by immersion in HISTO‐2 (H2‐30, Sigma‐Aldrich, MA, USA) for at least 48 h at room temperature with gentle shaking.

**TABLE 2:** Details of primary and secondary antibodies used in 3D immunofluorescent labelling.

Primary antibody	Company	Cat. no.	Dilution	Fixative	Secondary antibody	Dilution	Target
VWF	Dako	A0082	1 in 250	4% PFA	Anti‐rabbit AF594	1 in 500	Endothelial cells
Desmin	Dako	M0760	1 in 250	4% PFA	Anti‐mouse AF488	1 in 500	Smooth muscle cells
MYH11	Thermo Scientific	PA5‐82526	1 in 250	4% PFA	Anti‐rabbit AF594	1 in 500	Smooth muscle cells

### 2.7 Confocal Imaging

The 3D immunolabelled samples were placed in a glass‐bottom dish fitted with a polystyrene frame (220.220.042, IBL Baustoff+Labor GmbH, Austria) and a drop of Histo‐2 was added to keep the sample hydrated. The samples were then imaged using a Leica TCS SP8 laser scanning confocal microscope (Leica Biosystems, Nussloch, Germany) with a 10× objective, 256 × 256 pixel resolution, 4.6 μm pixel size and 3.76 μm *z*‐axis steps. The pinhole size was set to 1 Airy unit, frame average was set to 1, and line average was set to 2 using lasers emitting light at 405 nm (blue channel), 488 nm (green channel) and 561 nm (red channel). The images were visualised using Leica LAS X software (version 3.5.5) within the 3D module and reconstructed and analysed using Avizo. The reconstructed volume of immunolabelled microvasculature was measured using the volume fraction function in Avizo.

### 2.8 Pericyte Density Analysis

After imaging by confocal microscopy, the 3D immunolabelled samples were OCT embedded (*n* = 4; each age group), and 10 μm cryosections were cut. To visualise small vessel networks in SDFTs the sections were labelled with PDGFRB antibody, a pericyte cell marker, as described in Section 2.2. Images were captured using an Eclipse Ni‐E upright microscope with a 40× objective, and all the visible small vessels (capillaries, arterioles and venules) in each section were imaged to allow quantification of the total number. The pericyte density was calculated by normalising the number of PDGFRB positive vessels to the area of each section. The experiments were conducted in technical triplicate.

### 2.9 Measurement of Interfascicular Matrix Area and Width

The same samples used for pericyte density analysis (Section 2.8) were used to measure IFM area and width in young and old tendons (*n* = 4; each age group). DAPI was used to stain nuclei to allow identification of the IFM. Images were taken using an Eclipse Ni‐E upright microscope with a 40× objective, and IFM regions visible in each section were imaged. The area and width of each IFM region were measured using ImageJ (National Instruments, Austin, USA). The experiments were conducted in technical triplicate.

### 2.10 Statistical Analysis

All data are expressed as the mean ± standard deviation (SD), and all experiments were conducted using 4 different animals from each age group. A D'Agostino and Pearson test was used to determine if the data followed a normal distribution. The Welch's *T* test was performed when the data had a normal distribution, and the Mann–Whitney test or two‐way ANOVA was performed for the other data to calculate the differences (*p* < 0.05) between different sample groups using GraphPad Prism version 10.2.3 (La Jolla, CA, USA).

## 3 Results

### 3.1 Identification of Markers for Microvascular Cells in Equine Tendons Using scRNA‐Seq and 2D Immunofluorescence Staining

**FIGURE 1:**
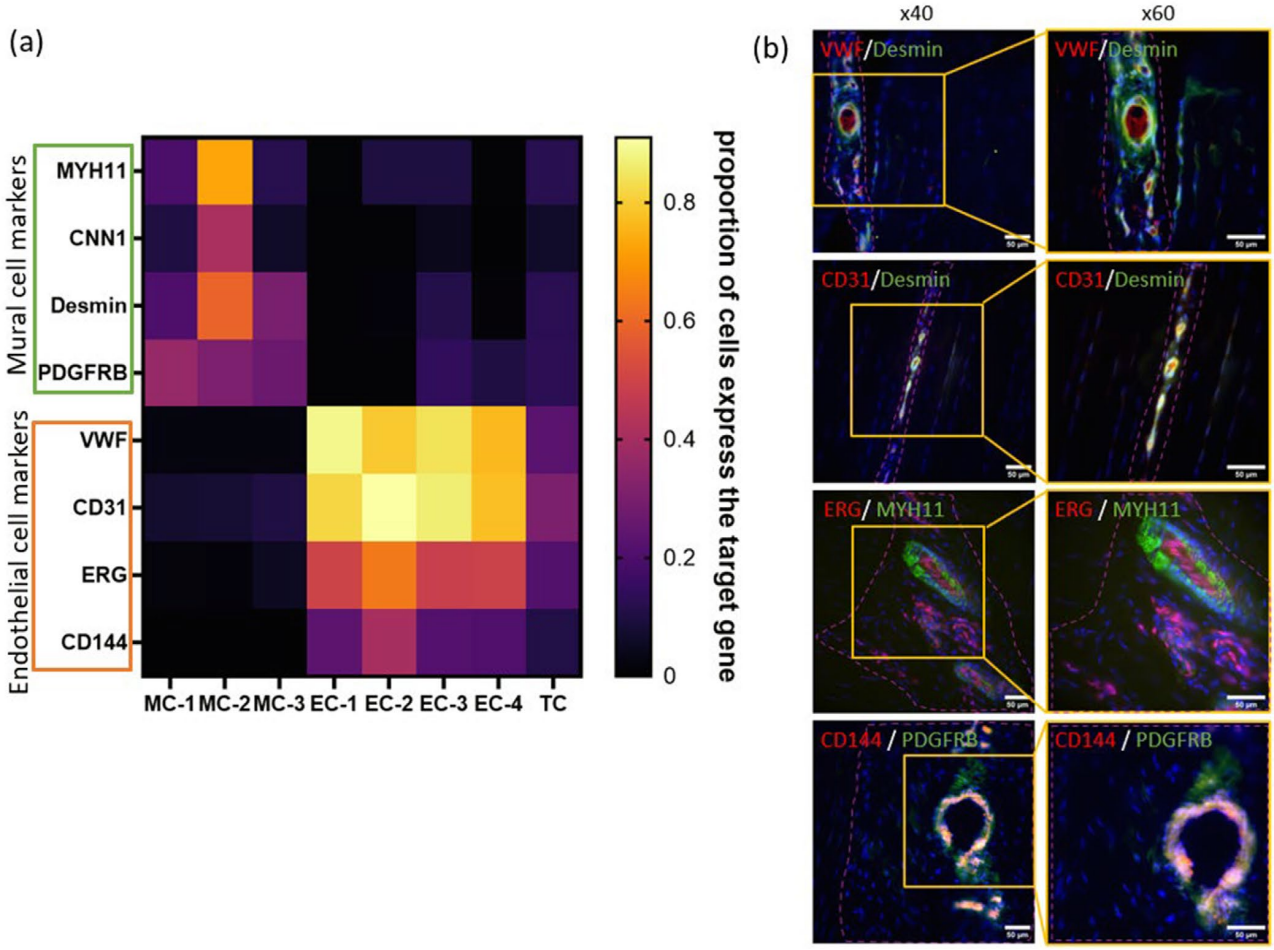
(a) A heatmap demonstrating the proportion of cells expressing vascular markers in mural cell (MC), endothelial cell (EC) and tenocyte (TC) clusters. (b) Representative immunofluorescence images of the tendon microvasculature in a young horse. Red: Endothelial cell markers; green: Mural cell markers; blue: Nuclei. Purple dashed lines demarcate the IFM. Scale bar is 50 μm.

To confirm the expression of vascular cell markers in SDFTs, a range of marker‐specific antibodies was used in this study, which were selected based on scRNA‐seq data obtained previously from equine tendons. As shown in the heatmap in Figure 1a, expression of the smooth muscle cell markers MYH11, calponin 1 (CNN1) and desmin was particularly elevated in one of the mural cell clusters (MCs), MC‐2, compared to the other MCs, endothelial cell clusters (ECs) and the tenocyte cluster (TC). The expression of the pericyte marker, PDGFRB, was elevated more in one particular MC (MC‐1) than the other MCs, ECs and TC. In addition, expression of the endothelial cell markers, VWF, CD31, ERG and CD144, was all higher in ECs than in MCs and the TC. Therefore, antibodies used thereafter in this study to identify proteins in the tendon microvasculature were selected on the basis of these gene changes.

Age‐associated changes in gene expression were assessed across individual cell clusters. The analysis did not show significant differences in the vascular marker expressions in MCs and ECs with advancing age, apart from desmin in MC‐2 and MYH11 in EC‐3. The average log_2_ fold change of desmin in MC‐2 (0.4784) and MYH11 in EC‐3 (2.4561) exhibited statistically significant age‐related upregulation of the genes and *p* values were 0.0004 and 0.0212, respectively (Table S1).

Immunofluorescence staining was performed on cryosections of SDFTs from young horses to characterise the vascular architecture and determine the spatial distribution of key vascular markers. All vasculature was localised to the IFM, with endothelial markers VWF, CD31, ERG and CD144 mainly localised to the inner layer of the vessels, and mural cell marker staining (Desmin, MYH11 and PDGFRB) surrounding the endothelial cells (Figure 1b). These findings provide a detailed spatial map of vascular cell marker distribution within the tendon, highlighting the IFM as the primary site of vascularisation.

### 3.2 μCT Revealed Decreased Vascular Volume, Diameter and Density in Aged Tendons

**FIGURE 2:**
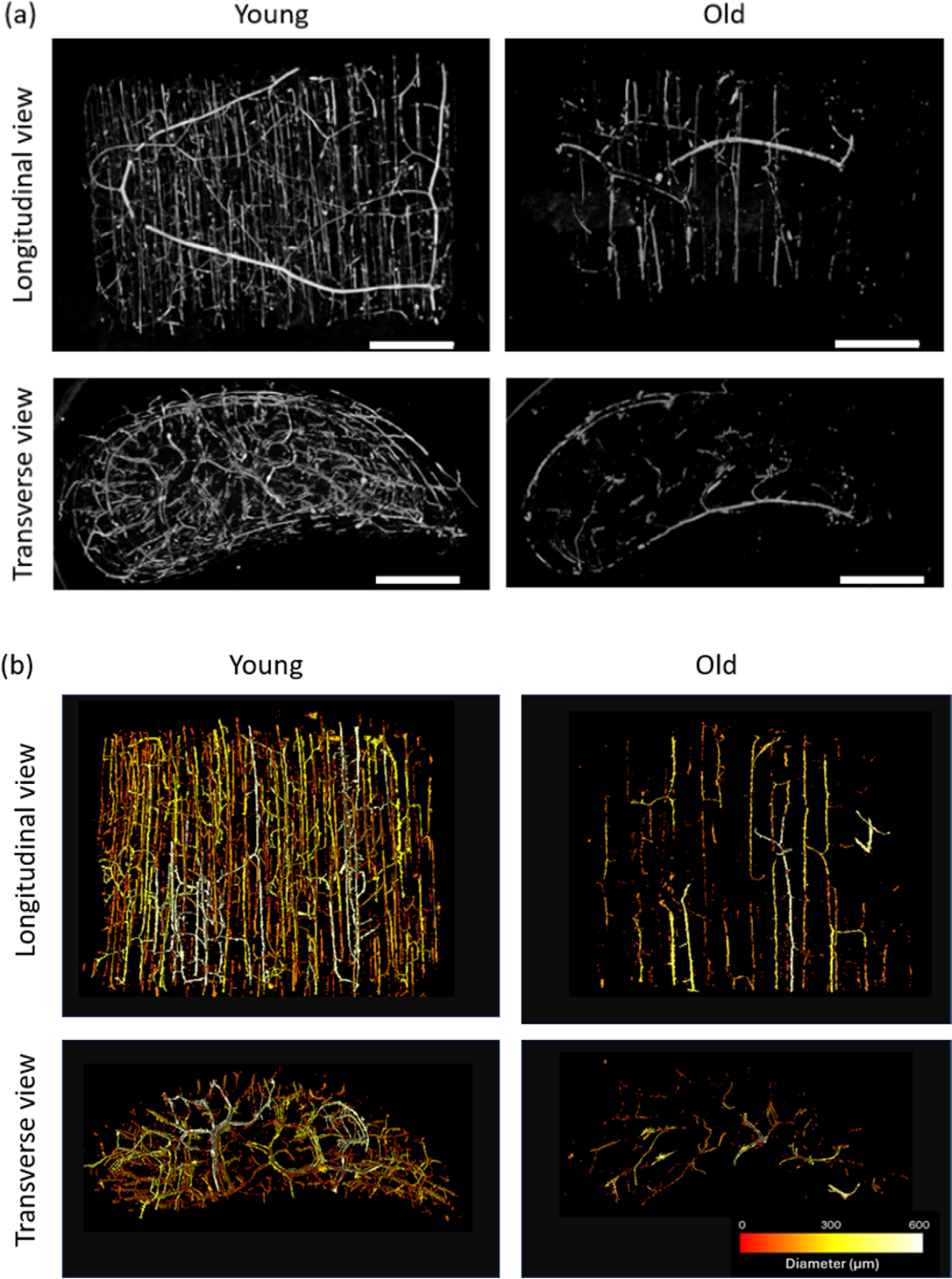
(a) Representative 3D reconstructed μCT images of young and old tendon microvasculature in epitenon and IFM in longitudinal and transverse views. Scale bar is 5 mm. (b) Representative 3D reconstructed μCT images of young and old tendon microvasculature in IFM colourised by vascular diameter. Red shows small vessels and white shows large vessels. Epitenon was removed from the images prior to the analysis.

Barium sulphate‐perfused SDFTs were imaged using μCT, which successfully visualised the SDFT vasculature (Figure 2a). The imaging revealed that young SDFTs exhibit a denser vascular network compared to old SDFTs. No evidence of barium sulphate leakage from the vessels was observed, indicating that all visualised structures represented tendon vasculature. Furthermore, when the images were colour‐coded according to vessel diameter, larger vessels were predominantly observed in the core of young SDFTs, whereas such large vessels were absent in older SDFTs (Figure 2b). However, the smallest detected vessel diameter was 37 μm with this technique, such that it was not suitable for visualising smaller vascular networks such as capillaries.

**FIGURE 3:**
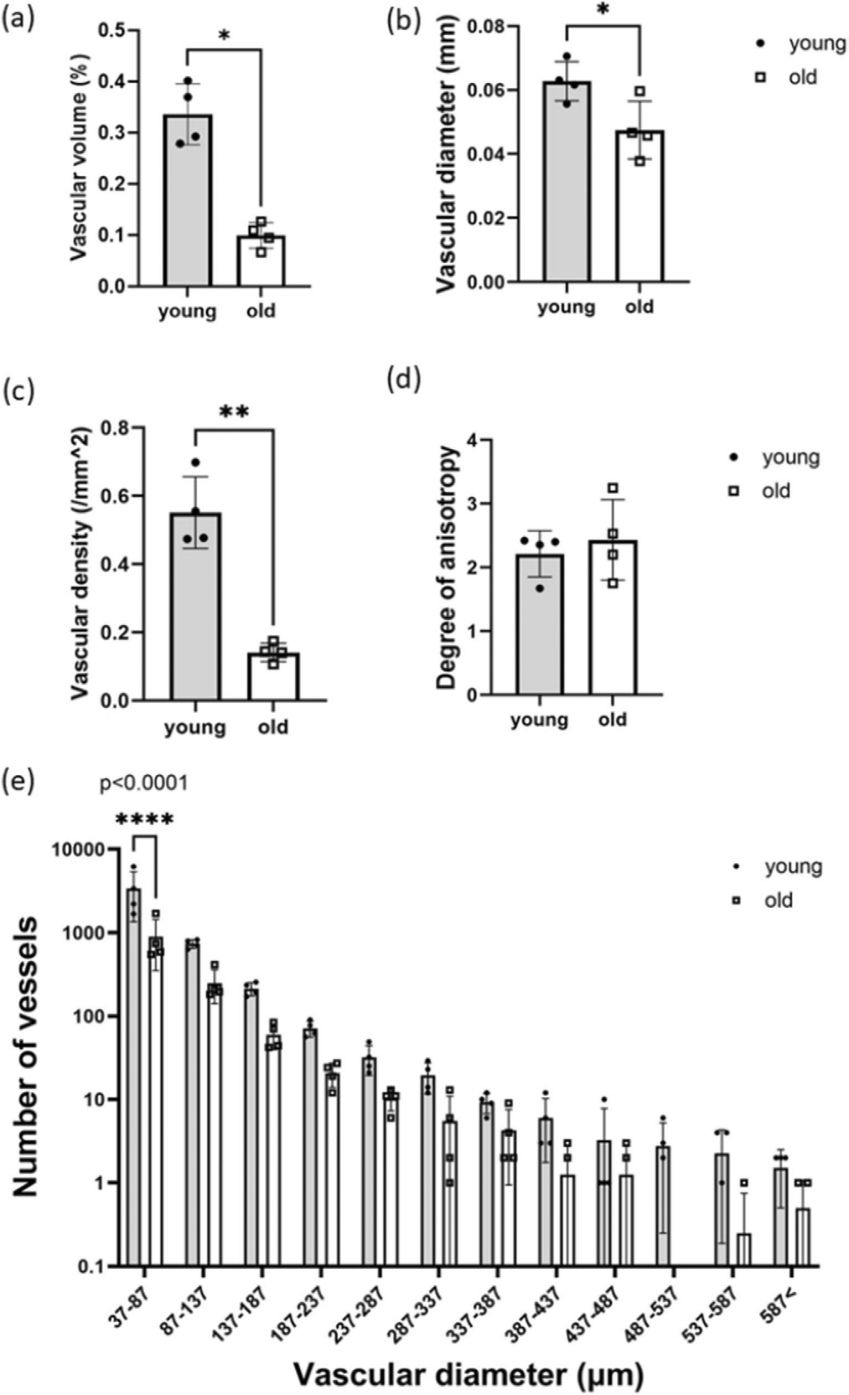
μCT image analysis shows a reduction in vasculature with age. (a) Vascular volume, (b) mean vascular diameter, (c) vascular density and (d) degree of anisotropy in young and old tendons. Mann–Whitney tests were used to calculate the significance between young and old tendons. (e) Histogram of vessel diameter in young and old tendons. Two‐way ANOVA followed by Šídák's multiple comparisons test was used to calculate the significance between the age groups. Due to the scale used (Log 10), data points with a value of zero are not displayed in the histogram. Data are presented as mean ± SD from analysis of 4 young and 4 old tendons: *p < 0.05 **p < 0.01 ****p < 0.0001.

Analysis of the μCT images revealed significant age‐related reductions in vascular volume, vessel diameter and density (Figure 3a–c). The mean vascular volume, vessel diameter and density were decreased by 70%, 30% and 74% respectively with age. In contrast, the degree of anisotropy, which measures the directional alignment of the vascular network, did not differ significantly with age (Figure 3d). The histogram of vessel diameter showed that old tendons have significantly fewer vessels with diameters between 37 and 87 μm compared to young tendons; however, there were no significant differences in the number of larger vessels (Figure 3e).

### 3.3 High Resolution Confocal Microscopy Demonstrated That Pericyte Density in Tendons Increases With Age

**FIGURE 4:**
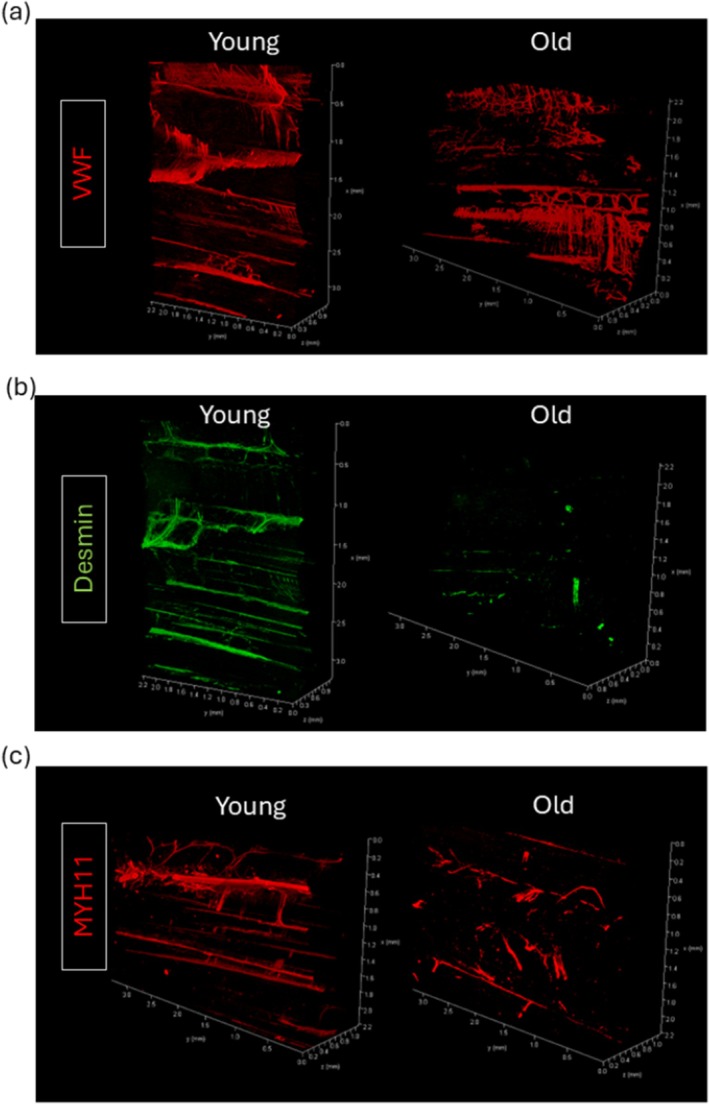
Representative reconstructed 3D immunolabelled images of young and old tendon microvasculature using (a) VWF antibody, (b) desmin antibody and (c) MYH11 antibody. Scales are in mm.

High‐resolution 3D imaging of the tendon vasculature was conducted using a combination of 3D immunolabelling and confocal microscopy, which allowed visualisation of smaller vasculature. The immunolabelling was successfully optimised to visualise endothelial and smooth muscle cell populations within the tendon microvasculature. VWF was used to identify endothelial cells (Figure 4a), while desmin and MYH11 were employed to label smooth muscle cells (Figure 4b,c). Overlaid images of VWF and desmin immunolabelling demonstrated that some VWF‐positive cells/vessels were surrounded by desmin‐positive cells, corresponding to larger vessels, whereas smaller VWF‐positive vessels lacked surrounding desmin‐positive cells, likely indicating the presence of smaller vessels or capillaries (Figure S2).

**FIGURE 5:**
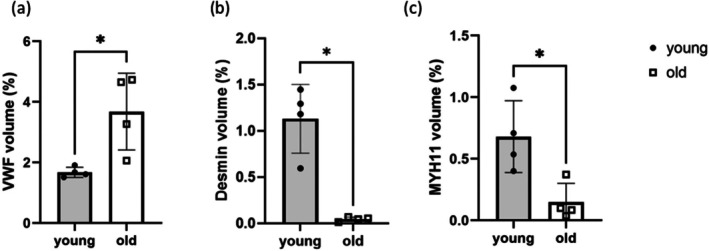
Immunolabelled 3D image analysis showed an increase in endothelial marker volume but decreases in smooth muscle cell marker volume. Immunolabelled volume for (a) VWF, (b) desmin and (c) MYH11 in young and old tendons. Data are presented as mean ± SD (*n* = 4). Mann–Whitney test was used to calculate the significance between young and old tendons. *p < 0.05.

The labelled volume was analysed to investigate age‐related alterations in endothelial and smooth muscle cells within the SDFT. Quantitative analysis revealed a significant increase of 220% in VWF‐positive volume with age (Figure 5a), suggesting an expansion of the endothelial cell population. Conversely, there were significant reductions in the volumes positive for desmin and MYH11, decreasing by 96% and 78%, respectively (Figure 5b,c), indicating a reduction in smooth muscle cell presence.

**FIGURE 6:**
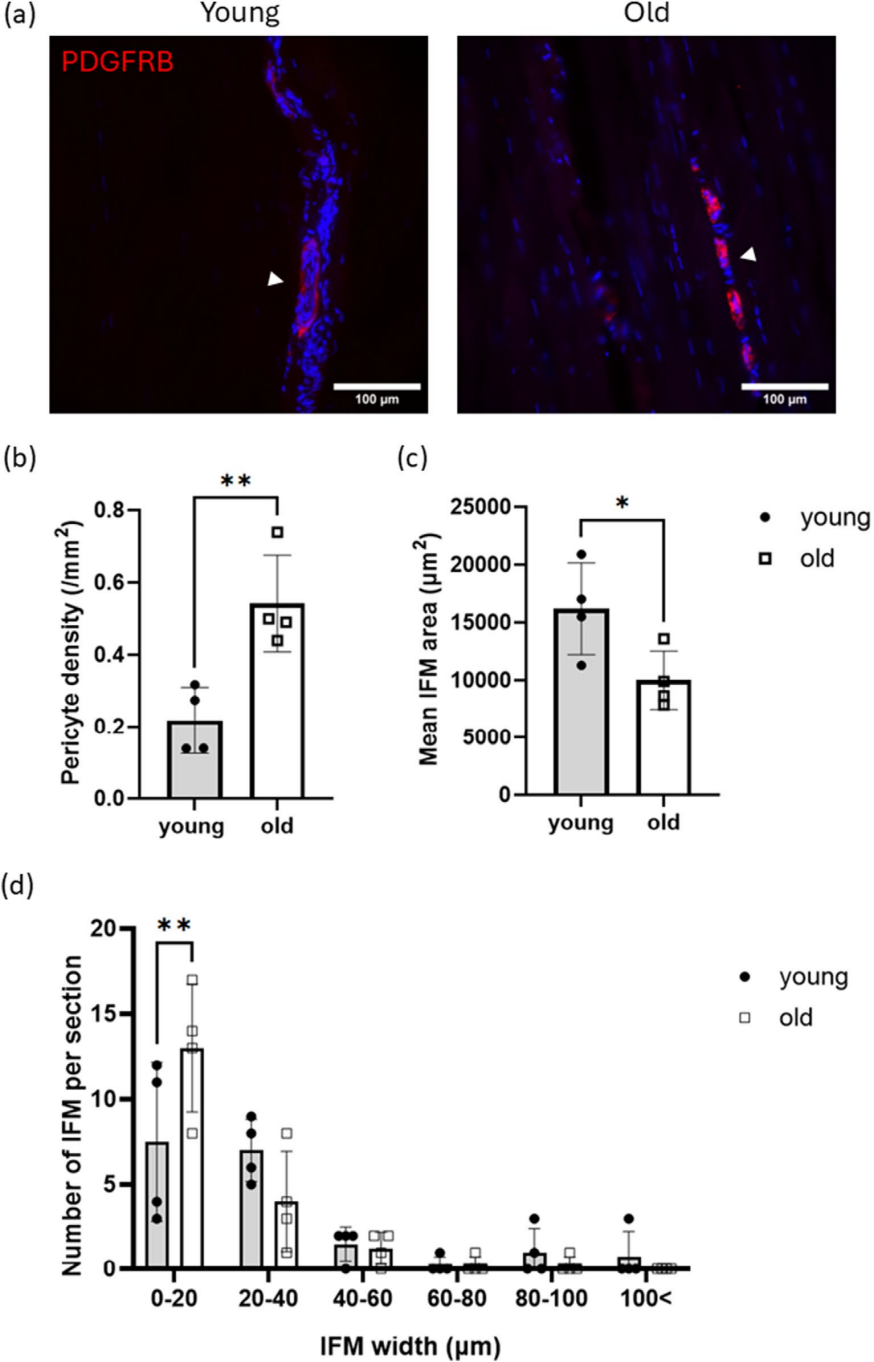
(a) Representative immunofluorescent images of pericytes (PDGFRB; red) in young and old tendons. White arrow heads demonstrate the capillaries. (b) Pericyte density (/mm^2^), (c) mean IFM area (μm^2^) and (d) histogram of number of IFM regions per section in young and old tendons are demonstrated. Data are presented as mean ± SD (*n* = 4). Welch's test was used to calculate the significance between young and old tendons. *p < 0.05 **p < 0.01.

To assess the density of small vessels in the SDFT, sections cut from the 3D‐immunolabelled samples were imaged using a higher magnification objective (×40) compared to the 3D imaging (×10). Immunolabelling with PDGFRB, a pericyte marker, successfully identified small vascular networks within the SDFT (Figure 6a). Quantitative analysis of pericyte density revealed a significant increase of 249% with age, implying an increase in pericyte density (Figure 6b). Additionally, the area and width of the IFM were measured (Figure 6c,d). The results demonstrated a significant age‐related reduction in IFM area, with older SDFTs exhibiting a greater proportion of thinner IFM regions compared to younger SDFTs, indicating that the IFM becomes thinner with ageing.

## 4 Discussion

This study comprehensively characterised ageing‐related alterations in the tendon vasculature for the first time, demonstrating that ageing causes a significant decrease in vascular volume, density and diameter of larger blood vessels; however, pericyte density showed an increase with age, highlighting a potential increased pericyte density with age that may indicate neo‐angiogenesis induction in aged tendons.

We reanalysed previous scRNA‐seq data from tendons from young and old horses (Zamboulis et al. 2024) and used the literature to identify a panel of markers for the endothelial and mural cells of the tendon vasculature. The results demonstrated that the endothelial cells are positive for VWF, CD31, CD144 and ERG, forming the inner layer of the vessels, and mural cells are positive for MYH11, desmin and PDGFRB, forming the outer layers of the vessels. In addition, differential expression of markers between different mural cell clusters indicates that MYH11 and desmin are specific markers of smooth muscle cells, present in the walls of arterioles and venules, and PDGFRB is a specific marker of pericytes, which surround endothelial cells in capillaries as well as in arterioles and venules.

We also determined if any of the genes used as markers for tendon vascular cells were differentially expressed with ageing. While most of the changes were not statistically significant, two mural cell markers (desmin and MYH11) showed a significantly increased expression with age but only in one of the clusters (MC‐2 and EC‐3 respectively). The age‐related gene expression changes of vascular markers in equine tendons did not follow the same trend as the protein expression, which showed decreases in vascular smooth muscle cell protein (MYH11 and desmin) and increases in endothelial cell (VWF) and pericyte proteins (PDGFRB) with age. While there is often poor correlation between mRNA and protein expression levels (Wang 2008), there is a significantly better correlation when the gene is differentially expressed (Koussounadis et al. 2015). However, transcriptome‐proteome decoupling has been shown to occur as a consequence of ageing (Llewellyn et al. 2023), which may explain the discrepancy between results at the gene and protein levels in the current study.

Mature tendon has historically been reported as relatively avascular (Ahmed et al. 1998; Schmidt‐Rohlfing et al. 1992). In the present study, however, μCT imaging of barium sulphate perfused tendons successfully visualised the tendon vasculature at high resolution, establishing a reliable method to quantify the tendon vasculature, and, in combination with immunolabelling, demonstrated a greater vascular network in young tendon, which localises to the epitenon and IFM. Despite this, our μCT system was unable to image the IFM region separately from the FM, preventing direct visualisation of vessel localisation within the IFM in 3D. Nevertheless, IFM localisation of tendon vasculature has been well documented in previous studies (Marr et al. 2023; Zamboulis et al. 2024), and is further supported by the 2D immunofluorescence imaging performed in this study.

There was a significant reduction in average vascular volume, diameter and density with increasing age, indicating a loss of the larger vessels (diameter ≥ 37 μm) that could be visualised using μCT. However, there was no significant difference in the degree of anisotropy, which measures the directional alignment of the vasculature, with age. These results indicate that while older tendons are less well vascularised, the organisation of the remaining vasculature remains unchanged. These findings are supported by previous studies showing a significant reduction in blood flow and vascular density in rodent and human tendons with ageing (Brewer 1979; Marqueti et al. 2018; Márquez‐Arabia et al. 2017; Rudzki et al. 2008). For example, previous studies using 2D histological analysis showed that vascular density in aged tendons decreases by approximately two‐thirds of that observed in young tendons (Marqueti et al. 2018; Márquez‐Arabia et al. 2017), comparable to the 74% decrease in vascular density reported in the current study. In addition, scRNA‐seq revealed that tendon microvascular cells were affected by ageing, with differentially expressed genes associated with senescence and inflammation (Zamboulis et al. 2024). The results of the 3D analysis techniques employed herein challenge the long‐standing dogma that tendon vascularity is unaffected by ageing (Gillis et al. 1997). However, the mechanisms driving the loss of tendon vasculature are still unclear and these should be elucidated in future studies.

Although the μCT imaging approach used in this study successfully showed larger blood vessel networks within the SDFTs, it is limited by its resolution. The chosen resolution of 10 μm, optimised for imaging the entire piece of tendon, was insufficient to capture the finer details of small capillaries. Higher resolution images were taken using smaller tendon pieces; however, small vessels/capillaries could not be visualised. This suggests that the barium sulphate gelatine solution did not penetrate through smaller vessels potentially due to the high viscosity of the solution. As a result, portions of the microvascular network may be missing from the reconstructions, which we acknowledge as a limitation of our study. Despite the limitation, the barium sulphate perfusion technique enabled specific visualisation of the tendon vasculature without enhancing contrast in other tendon structures.

Another limitation of this study is potential sample variability. The horse limbs were sourced from a commercial abattoir, and detailed information on the history of the horses, including exercise history, was unavailable. Exercise has been reported to influence vascular cell function and health, as well as ageing processes in musculoskeletal tissues such as bone and skeletal muscle in rodents (Kodama et al. 2004; Laufs et al. 2004; Park et al. 2025; Vezér et al. 2023). However, the effects of exercise on equine tendon vasculature are unknown. To minimise the sample variability, young and old horses were selected within a defined age range (2–5 years and 18–22 years respectively).

Additionally, while μCT provides detailed structural information, it does not offer cellular‐level insights, limiting the ability to assess cellular and molecular characteristics of the vasculature. Therefore, 3D immunolabelling and confocal microscopy were used to further investigate the tendon microvasculature. The combination of 3D immunolabelling and confocal microscopy allowed high‐resolution imaging (4.56 μm resolution), which allowed visualisation of smaller vessels, including capillaries, and overcame the limitation of μCT imaging. In addition, targeted labelling specific for endothelial cells using VWF, and for smooth muscle cells using MYH11 and desmin enabled cellular‐level analysis of the tendon microvasculature. The results showed significant decreases in MYH11 and desmin expression with age, which supports the data from the μCT image analysis; however, VWF volume was significantly elevated in old versus young tendons, suggesting an increase in the capillary network in aged tendons.

Assessment of the pericyte marker, PDGFRB and 2D immunolabelling were used to confirm the age‐related increase in the small vessel network. Although there are many well‐recognised regulators of angiogenesis, such as VEGF and FGF, their in vivo half‐lives are relatively short (5.37 and 7.6 h, respectively) (Beenken and Mohammadi 2009; Wang et al. 2025). Given that the samples used were sourced from a commercial abattoir and subjected to 4–5 h of transport prior to overnight cold storage and next day sample processing, measuring the expression of these growth factors would likely not accurately reflect the representation of angiogenic activity. Therefore, PDGFRB was utilised as a marker of pericytes to show small vessel formation. Using this approach, a significant increase in pericyte density with age was demonstrated, suggesting the possibility of enhanced angiogenic activity in aged tendons compared with young tendons. This may be a compensatory response to the loss of larger diameter blood vessels and suggests a dysregulation of vascular homeostasis with aged tendon. Consistent with this, increased neovascularisation has also been reported in tendinopathic tendons (Merkel et al. 2021). However, it is yet to be established whether this neo‐angiogenesis results in the formation of normal or dysfunctional blood vessels in tendons.

In normal adult tissues, angiogenesis (the formation of new blood vessels/capillaries from pre‐existing vasculature) is limited, occurring primarily during wound healing (Eelen et al. 2020; Han et al. 2022). Inflammation, which is associated with disease, injury and ageing, is a major driver of angiogenesis and could account in part for the neo‐angiogenesis evident in tendons from old horses (Granger and Senchenkova 2010). Hypoxia, a condition caused by low levels of oxygen in local tissues or the whole body, is another factor that induces angiogenesis and is associated with age‐related functional decline (Yeo 2019). Therefore, the increased pericyte density detected in the aged tendons suggests that the old tendons examined in this study may have experienced inflammation, potentially due to hypoxia, or previous micro‐damage that had been repaired. In future work, investigation of the history of micro‐damage by labelling for protein neo‐epitopes generated during microdamage, such as cartilage oligomeric matrix protein fragments, may shed some light on this since these are reported to be markers of tendon injury (Smith et al. 2020). In addition, future studies should investigate mechanisms that induce angiogenesis in aged tendons using in vitro vascularised tendon models incorporating induced senescence, injury and inflammation.

The images also revealed a significant age‐related reduction in IFM area, with aged tendons exhibiting a significantly higher number of thin IFM regions compared to young tendons, confirming earlier work (Thorpe et al. 2013). These findings suggest that both larger vessels shown by μCT (excluding capillaries) in tendon, and the IFM undergo structural shrinkage with ageing, which may have implications for tendon function and regeneration.

## 5 Conclusions

This study provides the first in‐depth characterisation of the tendon microvasculature and identifies a range of alterations in tendon vasculature with ageing, including structural changes using μCT imaging and cellular changes using immunolabelling and confocal microscopy. The μCT results showed significant reductions in vascular volume, diameter and density of larger blood vessels with age. However, immunolabelling revealed significant increases in endothelial cell marker and pericyte marker densities with age, suggesting that the formation of smaller vessels/capillaries may be induced in aged tendon. The induced angiogenesis may be due to inflammation caused by previous micro‐damage within the tendon, an area which requires further investigation. Identifying these age‐related alterations in tendon vasculature provides significant insight into tendon healing and regeneration, which may lead to the development of more effective therapeutics.

## Author Contributions

N.I. contributed to all aspects of this study, including research design, sample collection and processing, data acquisition and analysis, and writing/revision of the original manuscript. J.L. contributed to sample collection and processing. J.B. contributed to data analysis. D.E.Z. contributed to research design. E.J.T.F., C.P.D.W.‐J. and C.T.T. contributed to research design and interpretation of results and funding acquisition. All authors contributed to the editing of the paper.

## Conflicts of Interest

The authors declare no conflicts of interest.

## Supporting information


**Figure S1:** Representative 3D reconstructed μCT images of young tendon microvasculature in transverse view illustrating the segmentation process. The blue region represents the central part of the tendon, containing both the tendon fascicles and IFM, while the grey region represents the epitenon, which was excluded during segmentation for subsequent 3D image analysis. Scale bar is 5 mm.
**Figure S2:** Representative reconstructed and overlaid 3D immunolabelled images of young and old tendon microvasculature, stained with VWF and desmin antibodies. Scales are in mm.
**Table S1:** Average log2 fold change of vascular gene expression in mural cell clusters and endothelial cell clusters. Data w obtained from young (n = 4; age: 3–4 years) and old (n = 4; age: >17 years) horse SDFTs. A positive value indicates increased expression of the gene with age and a negative value decreased expression with age. *p < 0.05 ***p < 0.001.

## Data Availability

The data that support the outcomes of this study are available from the paper and supporting material. Upon request, raw data are available from the corresponding author.
