# Supplementary material for: Immunolabelling and Micro‐Computed Tomography Revealed Age‐Related Alterations in 3D Microvasculature of Tendons
Source: Aging Cell. 2025 Nov 18;25(1):e70293. doi: 10.1111/acel.70293 (PMC12740099; doi:10.1111/acel.70293)
Supplement: Supplementary file 1 — Figure S1: Representative 3D reconstructed μCT images of young tendon microvasculature in transverse view illustrating the segmentation process. The blue region represents the central part of the tendon, containing both the tendon fascicles and IFM, while the grey region represents the epitenon, which was excluded during segmentation for subsequent 3D image analysis. Scale bar is 5 mm. Figure S2: Representative reconstructed and overlaid 3D immunolabelled images of young and old tendon microvasculature, stained with VWF and desmin antibodies. Scales are in mm. Table S1: Average log2 fold change of vascular gene expression in mural cell clusters and endothelial cell clusters. Data w obtained from young (n = 4; age: 3–4 years) and old (n = 4; age: >17 years) horse SDFTs. A positive value indicates increased expression of the gene with age and a negative value decreased expression with age. *p < 0.05 ***p < 0.001. [file ACEL-25-e70293-s001.docx]

**Supplementary data**

*
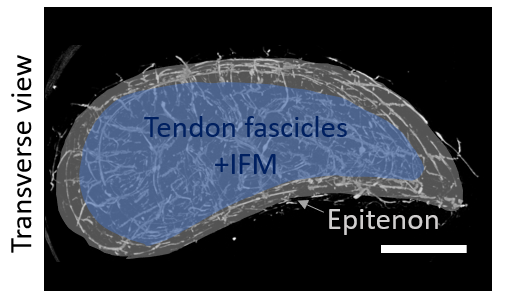
*

*Figure S1. Representative 3D reconstructed µCT images of young tendon microvasculature in transverse view illustrating the segmentation process. The blue region represents the central part of the tendon, containing both the tendon fascicles and IFM, while the grey region represents the epitenon, which was excluded during segmentation for subsequent 3D image analysis. Scale bar is 5mm.*

*Table S1. Average log_2_ fold change of vascular gene expression in mural cell clusters and endothelial cell clusters. Data w obtained from young (n=4; age: 3-4 years) and old (n=4; age: >17 years) horse SDFTs. A positive value indicates increased expression of the gene with age and a negative value decreased expression with age.* *p<0.05 ***p<0.001

|  | MYH11 | CNN1 | Desmin | PDGFRB | VWF | PECAM1 | ERG | CD144 |
| --- | --- | --- | --- | --- | --- | --- | --- | --- |
| MC-1 | 0.3391 | -0.1419 | 0.2574 | 0.2418 | -0.9789 | -0.1218 | 0.7857 | n/a |
| MC-2 | n/a | 0.2549 | 0.4784*** | 0.2761 | -0.5932 | 0.6027 | n/a | n/a |
| MC-3 | 0.5087 | -0.4368 | 0.2697 | 0.3528 | -0.5919 | 0.2519 | 0.6333 | n/a |
| EC-1 | -0.1589 | n/a | 1.0837 | n/a | -1.2501 | n/a | -0.8499 | n/a |
| EC-2 | 0.7950 | n/a | 0.3152 | 0.6707 | n/a | n/a | -0.1285 | n/a |
| EC-3 | 2.4561* | 0.7720 | 1.6834 | -0.1429 | -0.1573 | 0.3655 | 0.3391 | 0.8482 |
| EC-4 | n/a | n/a | 1.7302 | 1.2044 | -0.2420 | 0.3689 | 0.7178 | 1.0381 |

**
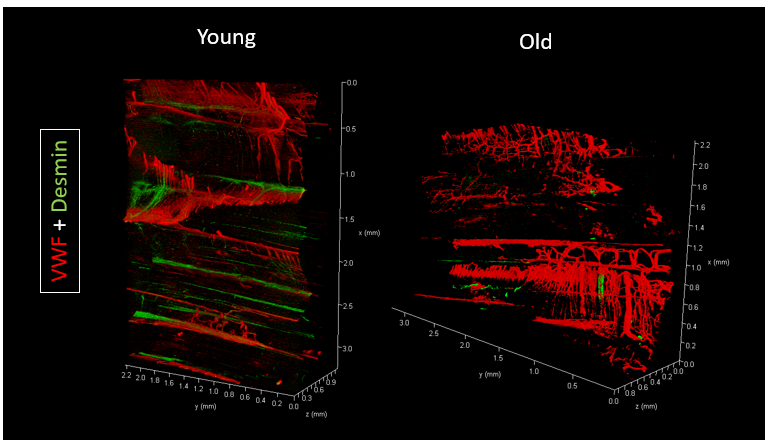
**

*Figure S2. Representative reconstructed and overlaid 3D immunolabelled images of young and old tendon microvasculature, stained with VWF and desmin antibodies. Scales are in mm.*
